# Ferroptosis as a promising therapeutic strategy for melanoma

**DOI:** 10.3389/fphar.2023.1252567

**Published:** 2023-09-19

**Authors:** Na Ta, Xiaodong Jiang, Yongchun Zhang, Hongquan Wang

**Affiliations:** ^1^ Department of Neurosurgery, The Affiliated Hospital of Chifeng University, Chifeng, China; ^2^ Department of Anatomy, College of Basic Medicine, Chifeng University Health Science Center, Chifeng, China; ^3^ Department of Oral and Maxillofacial Surgery, The Affiliated Hospital of Chifeng University, Chifeng, China; ^4^ Key Laboratory of Cancer Prevention and Therapy, National Clinical Research Center for Cancer, Tianjin’s Clinical Research Center for Cancer, Department of Pancreatic Cancer, Tianjin Medical University Cancer Institute and Hospital, Tianjin, China

**Keywords:** malignant melanoma, ferroptosis, ferroptosis inducer, small molecules compounds, dynamic therapy, nanomaterial

## Abstract

Malignant melanoma (MM) is the most common and deadliest type of skin cancer and is associated with high mortality rates across all races and ethnicities. Although present treatment options combined with surgery provide short-term clinical benefit in patients and early diagnosis of non-metastatic MM significantly increases the probability of survival, no efficacious treatments are available for MM. The etiology and pathogenesis of MM are complex. Acquired drug resistance is associated with a pool prognosis in patients with advanced-stage MM. Thus, these patients require new therapeutic strategies to improve their treatment response and prognosis. Multiple studies have revealed that ferroptosis, a non-apoptotic form of regulated cell death (RCD) characterized by iron dependant lipid peroxidation, can prevent the development of MM. Recent studies have indicated that targeting ferroptosis is a promising treatment strategy for MM. This review article summarizes the core mechanisms underlying the development of ferroptosis in MM cells and its potential role as a therapeutic target in MM. We emphasize the emerging types of small molecules inducing ferroptosis pathways by boosting the antitumor activity of BRAFi and immunotherapy and uncover their beneficial effects to treat MM. We also summarize the application of nanosensitizer-mediated unique dynamic therapeutic strategies and ferroptosis-based nanodrug targeting strategies as therapeutic options for MM. This review suggests that pharmacological induction of ferroptosis may be a potential therapeutic target for MM.

## 1 Introduction

Malignant melanoma (MM) is one of the most lethal and aggressive types of skin cancer and is associated with the highest rates of mutation and treatment resistance. It is responsible for the majority of skin-related cancer mortality worldwide, especially in its metastatic form (Anestopoulos et al., 2022; Wagstaff et al., 2022). The incidence of melanoma has been increasing worldwide (Tucker, 2009; Carr et al., 2020; Forsea, 2020; Bolick and Geller, 2021; Memon et al., 2021; Saginala et al., 2021). Both genetic and environmental risk factors have been reported to be associated with the onset of melanoma, with ultraviolet (UV) radiation exposure being the most prominent factor, especially in fair-skinned populations. Studies have revealed that *BRAF* (B-Raf proto-oncogene, serine/threonine kinase), *KRAS* (Kirsten rat sarcoma), *NRAS* (neuroblastoma RAS viral oncogene homolog), *HRAS* (Harvey Rat sarcoma viral oncogene), *CDKN2B* (Cyclin dependent kinase inhibitor 2B)*, PTEN* (phosphatase and the tensin homolog deleted on chromosome 10), *TERT* (telomerase reverse transcriptase), and *p53* are the most commonly mutated genes in MM progression, which can potentially cause resistance to targeted therapy. Moreover, *BRAF* mutations occur in approximately 60.0% of MM cases (Anestopoulos et al., 2022).

MM arises solely from melanocytes that is primarily localized in the skin, or may also occur in mucous membranes (the digestive, respiratory, and genitourinary tracts), the eye, and even in the leptomeninges (Thornton et al., 1988; Abdullah and Keczkes, 1989; Nicolaides et al., 1995; DeMatos et al., 1998). Due to its ability to spread and metastasize rapidly, MM is more dangerous than other skin cancers if not removed at an early stage (Pastwińska et al., 2022). Immune checkpoint inhibitors (ICI) and targeted therapy are the mainstay for the treatment of metastatic MM (Reuben et al., 2017), while surgical resection is the major treatment option for the localized melanoma (Tyrell et al., 2017). Treatment for metastatic melanoma is very challenging, and chemotherapy regimens have been identified as an important therapeutic option in managing patients with metastatic and/or advanced-stage MM. The most important challenge with anti-MM therapies face is that melanoma cells intrinsically evades cell death-induced by anticancer drugs (Abildgaard and Guldberg, 2015; Kalal et al., 2017). Identification of the molecular mechanisms of chemoresistance is vital for the development of effective therapeutic strategies to overcome drug resistance.

Ferroptosis, which is defined as an iron-dependent form of regulated cell death (RCD) driven by lipid peroxidation (LPO) in cellular membranes, has been considered to be a potential therapeutic strategy against tumors, including MM, since its discovery in 2012 (Lei et al., 2021; Wang L. et al., 2023). Increasing evidence over the last decade has shown that activation of ferroptosis suppresses the development of many chemotherapy-resistant cancers (Lu et al., 2017; Guo et al., 2018), and that targeting ferroptosis is a promising treatment strategy for MM. However, a greater and better understanding of the molecular mechanisms underlying the initiation and propagation of ferroptosis as well as the resistance to this form of RCD in MM is needed.

In this review, we have summarized the core mechanisms of ferroptosis in MM and its potential effects in MM treatment. We emphasize the roles of emerging types of small molecules inducing ferroptosis pathways by boosting the antitumor activity of BRAFi and immunotherapy and delineate their beneficial effects in treating MM. In addition, we have summarized the application of nanosensitizer-mediated unique dynamic therapeutic strategies and ferroptosis-based nanodrug targeting strategies as therapeutic options for MM. We also highlight future research perspectives for ferroptosis in MM, which could help enhance the understanding of this topic. This review suggests that pharmacological induction of ferroptosis as a potential therapeutic regimen for MM.

## 2 Materials and methods

Searches were conducted in the PubMed database for the period between January 2012 and August 2023. The keyword used was “Melanoma” AND “Ferroptosis” Only English reports were considered. Reference lists of original research studies were manually searched. The manuscripts of all potentially relevant research studies that investigate the association between ferroptosis and Melanoma identified during the search of abstracts were then retrieved and reviewed. The MEDLINE search resulted in 145 articles. Of them, 13 were excluded because they were Review Article. The remaining 132 articles were evaluated, from which we exam 1) The role of ferroptosis in melanoma; 2) Induction of ferroptosis as a novel approach to treat melanoma.

## 3 A concise overview of ferroptosis

**FIGURE 1:**
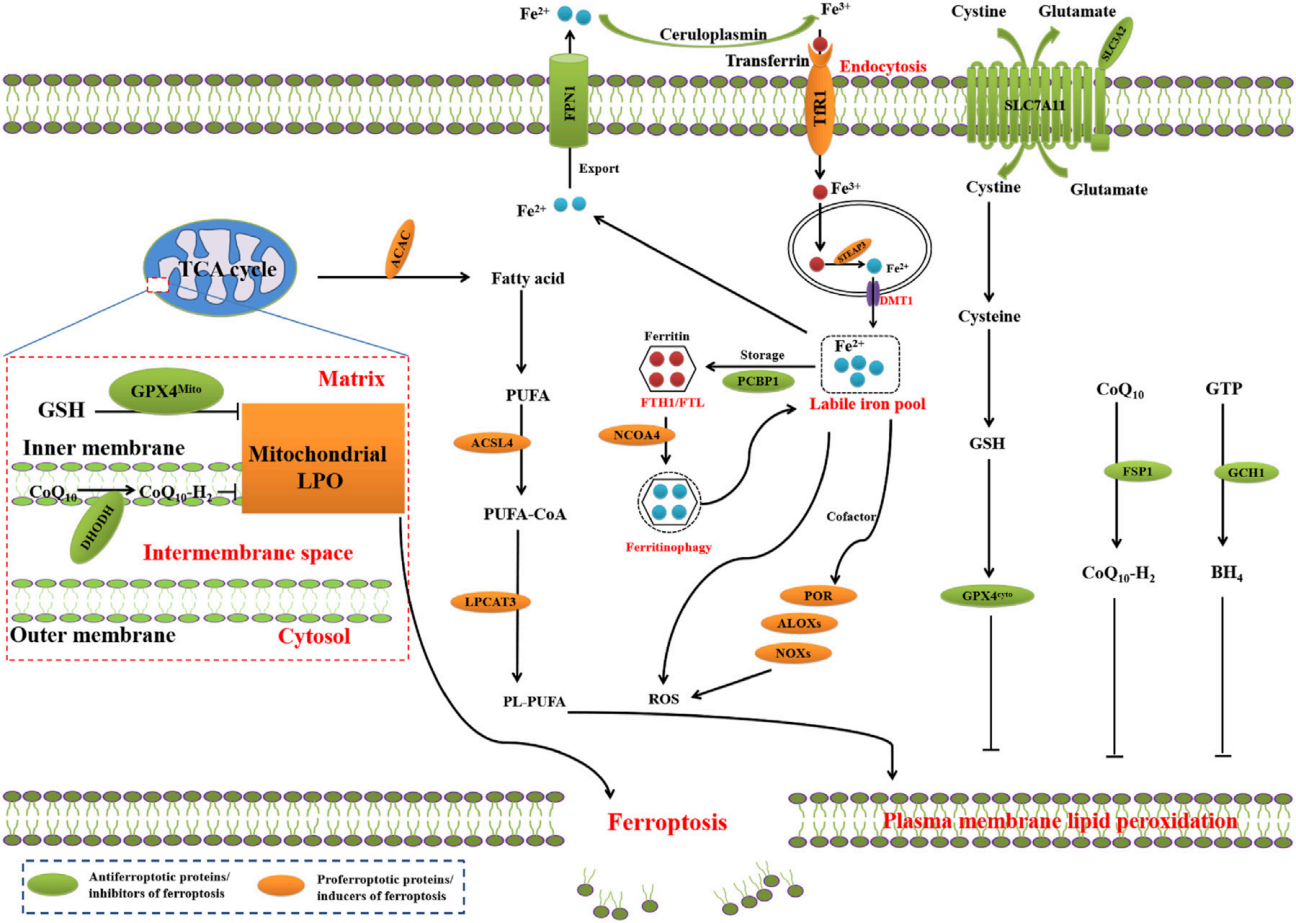
Core mechanisms of ferroptosis. The core of ferroptosis initiation is iron-dependent lipid peroxidation of polyunsaturated fatty acid (PUFA)-containing phospholipids (PUFA-PLs). When the ferroptosis-promoting factors (or Ferroptosis prerequisites) exceeding the buffering capability of cellular antioxidant systems (or ferroptosis defence systems), lethal accumulation of lipid peroxides on cellular membranes lead to membrane rupture, resulting in ferroptosis-related cell death. The ferroptosis-promoting factors consist of PUFA-PL synthesis and peroxidation, iron metabolism among others. Cells have evolved at least four ferroptosis defence systems, which includes GPX4/xCT system, the FSP1/CoQH_2_ system, the DHODH/CoQH_2_ system, and the GCH1/BH_4_ system, with different subcellular localizations to detoxify lipid peroxides and thus protect cells against ferroptosis. The cytosolic GPX4 (GPX4^cyto^) cooperates with FSP1 on the plasma membrane (and other non-mitochondrial membranes) and mitochondrial GPX4 (GPX4^mito^) cooperates with DHODH in the mitochondria to neutralize lipid peroxides. ACSL4 and LPCAT3 mediate the synthesis of PUFA-PLs, which are susceptible to LPO through both non-enzymatic and enzymatic mechanisms. Iron initiates the non-enzymatic Fenton reaction and acts as an essential cofactor for ALOXs and POR, which promote LPO. When ferroptosis-promoting factors significantly exceed the detoxification capabilities of ferroptosis defence systems, an excessive and lethal accumulation of lipid peroxides on cellular membranes result in membrane rupture and trigger ferroptosis-mediated cell death.

Ferroptosis, a term coined in 2012, refers to an new form of RCD driven by an iron-dependent LPO on cellular membranes or organelles (Dixon et al., 2012). The core step in ferroptosis is iron-catalyzed peroxidation of PL-PUFAs. When ferroptosis-promoting factors exceed the buffering capability of ferroptosis-defense systems, lethal lipid peroxides accumulates on cellular membranes, leading to membrane rupture and ferroptosis-mediated cell death (Qiu et al., 2020; Wang Y. et al., 2023) (Figure 1).

Membrane LPO, the core mechanism underlying ferroptosis, is a radical-mediated chain reaction involving a series of chemical reactions among iron, oxidizable lipids, and molecular oxygen (O_2_), leading to the incorporation of O_2_ into lipids (Conrad and Pratt, 2019; Dixon and Pratt, 2023). The polyunsaturated fatty acid-containing phospholipids (PL-PUFAs) are LPO substrates during ferroptosis (Conrad and Pratt, 2019; Hadian and Stockwell, 2020).

The critical mediators of PL-PUFAs synthesis include acyl-coenzyme A (CoA) synthetase long chain family member 4 (ACSL4) and lysophosphatidylcholine acyltransferase 3 (LPCAT3) (Doll et al., 2017; Kagan et al., 2017). ACSL4 induces the ligation of PUFAs with CoA to produce acyl-CoA (ACA), which can be re-esterified by LPCATs to produce PL-PUFAs. Under the help of acetyl-CoA carboxylase, ACA functions as the building block for PUFA synthesis (Hadian and Stockwell, 2020). LPO of PL-PUFAs is primarily catalyzed by iron-mediated Fenton reaction-driven non-enzymatic autoxidation (Gaschler and Stockwell, 2017; Shah et al., 2018; Conrad and Pratt, 2019). The enzymatic reactions mediated by arachidonate lipoxygenase (ALOX) or cytochrome P450 oxidoreductase (POR) under the influence of labile iron have also been shown to promote LPO (Yang et al., 2016; Wenzel et al., 2017; Zou et al., 2020; Koppula et al., 2021; Yan et al., 2021). Membrane-associated PL-PUFAs, labile iron, and POR or ALOXs undergo peroxidation reaction using O_2_ to generate lipid peroxides, PL-PUFA-OOH (Hadian and Stockwell, 2020; Zou et al., 2020). In the last step of ferroptosis, LPO or its secondary products, including 4-hydroxy-2-nonenal (4-HNE) or malondialdehyde (MDA), lead to pore formation in plasma and organelle membranes to mediate ferroptosis-related cell death.

## 4 The role of ferroptosis in melanoma

### 4.1 The metabolic switch dictates the vulnerablity of melanoma to ferroptosis

Mutated oncogenes in cancer cells rewire cellular metabolism networks to meet their increased demand for energy and nutrients (Martinez-Outschoorn et al., 2017; Wolpaw and Dang, 2018); however, this metabolic reprogramming in cancer cells often creates new metabolic liabilities, thereby making cancer cells, including MM, uniquely vulnerable to ferroptosis. The mutated proto-oncogene B-Raf (BRAF) negatively modulates oxidative metabolism in the cancer cells of MM (Haq et al., 2013), while BRAF inhibitors (BRAFi) increase the dependence of melanoma cells on oxidative phosphorylation (OXPHOS) (Haq et al., 2013; Schöckel et al., 2015). The sensitivity of cancer cells with OXPHOS^high^ to chemotherapy has been shown to depend on the accumulation of ROS and may be potentially initiated by the induction of ferroptosis (Gentric et al., 2019). This result suggests that the BRAFi-mediated metabolic switch can make melanoma cells sensitive to ferroptosis inducers, and that SCL7A11 may be a biomarker of the vulnerability of metastatic MM cells to ferroptosis (Gagliardi et al., 2019). Accordingly, trametinib or vemurafenib decrease the expression of SLC7A11 in melanoma cells bearing BRAF^V600E^ mutation (Osrodek et al., 2019).

Studies have also shown lipid metabolism reprogramming occurred in vemurafenib-treated melanoma cells, such as accumulation of PUFAs (Talebi et al., 2018). As an inhibitor targeting the mitochondrial complex I that impairs OXPHOS, BAY 87-2243 combined with vemurafenib inhibits tumor growth of melanoma *in vivo* (Schöckel et al., 2015), partially by inducing ferroptosis (Basit et al., 2017). The metabolic switch toward OXPHOS in melanoma cells can be further complicated by phenotypic alterations, i.e., bearing an acquired resistance to BRAFi and MEK inhibitors (MEKi), such as trametinib, cobimetinib, or binimetinib. Increased dependence on the glutamine metabolism has been shown to be related to acquisition of resistance since the resistance of melanoma cells to BRAFi promotes the synthesis of glucose-derived glutamate and an increase in GSH content (Khamari et al., 2018).

### 4.2 Ferroptosis evasion fuels tumors in MM

Inhibition of the synthesis of PL-PUFAs and LPO is one of mechanisms by which MM evades ferroptosis and enhances tumor development and metastasis. Recent studies have shown that evasion of ferroptosis (resistance to ferroptosis) through modulation of fatty acid metabolism accounts for cancer metastasis in MM. Before systemic metastasis through the blood, melanomas generally regionally metastasize through the lymphatic system. Melanoma cells in the lymphatic environment can evade ferroptosis, which enhances metastasis through the blood pathway (Ubellacker et al., 2020). The lymphatic environment, which has low levels of free iron as well as GSH and abundant levels of oleic acid (OA, a MUFA) in the lymphatic fluid, enhances evading ferroptosis *in vivo* (Ubellacker et al., 2020). OA suppresses ferroptosis in melanoma cells through MUFA-PL synthesis mediated by ACSL3, which displaces PUFAs from PLs (Ubellacker et al., 2020). Meanwhile, this lymphatic environment-mediated ferroptosis resistance increases subsequent survival of cancer cells during metastasis through the blood (Ubellacker et al., 2020).

Upregulation of ferroptosis defenses is another mechanism of MM tumors to evade ferroptosis, leading to promote tumorigenesis and metastasis. The Nrf2 functions as a master regulator of antioxidant defense and regulates the genes involved in GPX4-GSH-mediated ferroptosis defense, thereby promoting resistance to ferroptosis (Kerins and Ooi, 2018; Dodson et al., 2019; Kajarabille and Latunde-Dada, 2019; Qu et al., 2020; Lei et al., 2022). Strong activation of Nrf2 leads to an increased activity of pentose phosphate pathway that is implicated in the regeneration of GSH, and upregulation of SLC7A11 expression, which confers ferroptosis evasion and promotes tumor growth in drug-resistant MM cells (Khamari et al., 2018).

### 4.3 GPX4-dependent persister state confers therapy resistance

Increasing evidence has underscored the role of non-mutational mechanisms that confer resistance to cancer cells (Boumahdi and de Sauvage, 2020). Epithelial carcinoma cells can dedifferentiate to adopt a mesenchymal or mixed epithelial-mesenchymal (EM) phenotype, which is named as epithelial-mesenchymal plasticity (EMP) (Williams et al., 2019; Yang J. et al., 2020; Lambert and Weinberg, 2021). EMP is one of type of cancer cell plasticity that defines the ability of cancer cells to undergo dynamical and reversible changes between distinct phenotypical states, leading to the acquisition of cancer stemness properties, eventually resulting in resistance to therapy (Boumahdi and de Sauvage, 2020). Cancer cells with a mesenchymal state that usually become resistance to conventional therapies (i.e., apoptosis inducers) are strongly dependent on GPX4, which is related to upregulated expression of zinc finger E-box binding homeobox 1 (ZEB1) (Krebs et al., 2017). ZEB1 is a lipogenic factor and driver of epithelial-to-mesenchymal transition (EMT). Cancer cells in the mesenchymal state show enhanced PUFA-PL synthesis, possibly resulting from ZEB1-mediated upregulation of PPARγ, which ia a major regulator of lipid metabolism in liver (Viswanathan et al., 2017). High levels of ZEB1 increases cellular sensitivity to ferroptosis (Viswanathan et al., 2017). The enhanced PUFA-PL levels in MM cells can detoxify lipid peroxides dependent on GPX4 for survival, leading to the high vulnerability of these cancer cells to ferroptosis (Viswanathan et al., 2017). Persistent drug-resistant melanoma cells in a mesenchymal-like state have been shown to be highly vulnerable to GPX4 inhibition (Xu et al., 2019). GPX4 dependency makes melanoma cells derived from drug-resistant patients reliant on transforming growth factor beta (TGF-β) (Viswanathan et al., 2017). In addition, ablation of GPX4 induces chemoresistant A375 melanoma cell death, which can be reversed by ferrostatin-1, a ferroptosis inhibitor (Hangauer et al., 2017). In combination with dabrafenib and trametinib, ferrostatin-1 enhances tumor growth of xenografted mice bearing A375^GPX4−/−^ cells, while ferrostatin-1 withdrawal results in inhibiting the growth of GPX4^−/−^ tumors (Hangauer et al., 2017).

### 4.4 Cellular dedifferentiation status correlates with melanoma ferroptosis sensitivity

Cancer cellular dedifferentiation can drive cancer cell resistance to targeted therapy (Gupta et al., 2019). Particularly, MM cells highly maintain a plastic phenotype, possibly as a result of their origin from the neural crest, which contains a transient stem-cell-like embryonic cell population that can differentiate into various tissue types, including glia, neurons, or cartilage and endocrine cells (Sauka-Spengler and Bronner-Fraser, 2008). Dedifferentiation confers MM resistance to both inhibitors of BRAF and MAPK that are associated with loss of melanoma-specific transcription factor (MITF) (Khamari et al., 2018; Tsoi et al., 2018), which is the master regulator of melanocyte differentiation (Müller et al., 2014; Hugo et al., 2015). Furthermore, MITF downregulation has been shown to promote tumor growth and enhance immune evasion by facilitating the recruitment of immunosuppressive myeloid immune cells into the tumor microenvironment (Riesenberg et al., 2015). The sensitivity of cancer cells to ferroptosis can be dictated by dedifferentiation status, with a negative correlation between differentiation status and the vulnerability to ferroptosis in MM. BRAFi resistance-induced dedifferentiation confers the sensitivity of MM to RSL3 and erastin-induced ferroptosis. Meanwhile, GSH levels have been shown to be significantly related to the cell dedifferentiation stage (Tsoi et al., 2018), implying that GSH functions as a metabolic link between sensitivity to ferroptosis inducers and drug-associated dedifferentiation; this was confirmed by evidence showing that GSH supplementation can inhibit ferroptosis in MM (Tsoi et al., 2018). The cerebellar degeneration-related 1 antisense (CDR1as) functions as a new marker for differentiation status of MM cell, and ablation of CDR1as leads to the metastatic potential of MM (Hanniford et al., 2020). MM cells with high expression of CDR1as, which are marked by low level of microphthalmia-associated transcription factor and high level of the receptor tyrosine kinase AXL, have more sensitivity to inhibition of GPX4, thus inducing ferroptosis (Hanniford et al., 2020).

### 4.5 Other regulators of ferroptosis in MM

Several regulators working as non-canonical oncogenes are involved in the resistance of MM cells to ferroptosis. Erastin, a ferroptosis inducer, can upregulate the expression of oncogene NEDD4, a ubiquitin ligase(Wang et al., 2007; Yang Y. et al., 2020). Erastin induces ferroptosis through decreasing VDAC2/3 expression in a NEDD4-dependent manner, thereby inhibiting erastin-induced ferroptosis (Yang J. et al., 2020). The calcium/calmodulin dependent protein kinase 2 (CAMKK2), which plays a vital role in regulating intracellular calcium levels and signaling pathways, dictates the sensitivity of MM cells to ferroptosis (Wang H. et al., 2022). CAMKK2 negatively regulates ferroptosis through AMPK-dependently activating Nrf2 and suppressing LPO. CAMKK2 inhibition boosts the efficacy of anti-PD-1 immunotherapy and ferroptosis inducers by promoting ferroptosis through inhibition of the AMPK-Nrf2 pathway (Wang M. et al., 2022). These results suggest that targeting CAMKK2 can serve as a potential regimen for MM treatment by increasing the efficacy of ferroptosis inducers and immunotherapy. GPX4 has also been reported to support the activation of regulatory T (Treg) cells and prevent them from undergoing ferroptosis to suppress antitumor immunity in MM (Xu et al., 2021). T cell receptor/CD28 co-stimulation disturbs immune homeostasis devoid of affecting the survival of Treg cells in the steady state in GPX4-deficiency regulatory T cells (Treg), resulting in abberant accumulation of lipid peroxides, thereby leading to ferroptosis in Treg cells (Xu et al., 2021). Blocking iron availability and neutralizing lipid peroxides inhibits ferroptosis in GPX4-deficient Treg cells. GPX4 deficiency in Treg cells increases the production of mitochondrion-derived superoxide and interleukin-1β that promotes T helper 17 responses, thereby repressing tumor growth and concomitantly potentiating antitumor immunity (Xu et al., 2021). These results establish the vital role of GPX4 in inhibiting the ferroptosis of activated Treg cells, providing a potential therapeutic regimen to improve MM treatment (Xu et al., 2021). High expression of AXL in the majority of melanoma lymph node metastases limits treatment efficacy by promoting MM cell ability to a more aggressive mesenchymal phenotype switch from epithelial(Nyakas et al., 2022). The phospholipase A2 group VI (PLA2G6) was markedly upregulated in MM, and PLA2G6 silencing dramatically inhibited cell proliferation, migration and invasion associated with the ferroptosis (Wang S. et al., 2022). RSL3 and Erastin, the two ferroptosis inducers, can upregulate iron metabolism proteins, including transferrin receptor (TfR), ferritin heavy chain 1 (FTH1), and ferroportin (FPN) by inducing iron regulatory protein1 (IRP1) in MM cells. Ablation of IRP1 inhibits the erastin- and RSL3-induced ferroptosis. Overexpression of TfR and silencing FPN and FTH1 in IRP1 knockdown MM cells significantly enhances the ferroptosis induced by erastin and RSL3. These results suggest that IRP1 promotes erastin- and RSL3-mediated ferroptosis by regulating iron homeostasis (Yao et al., 2021). Sterol regulatory element-binding protein 2 (SREBP2), the master lipogenic regulator, directly upregulates the expression of the intracellular iron carrier transferrin (TF), which reduces the iron and LPO content to suppress ferroptosis and enhance the survival of circulating tumor cells (CTCs) and drug resistance (Hong et al., 2021). Increased lipogenesis mediated by SREBP2 directly upregulates the TF, reducing intracellular LIP, ROS, and LPO and thereby rendering MM resistance to ferroptosis inducers. This crosstalk between the lipogenic pathway and iron homeostasis accounts for therapeutic resistance and CTC-mediated tumorigenesis (Hong et al., 2021). Arginase 2 (Arg2) negatively regulates sorafenib-mediated ferroptosis in MM (Yu et al., 2022). Sorafenib induces melanoma cell death through decreasing expression of Arg2. Silencing Arg2 increases LPO and decreases the Akt phosphorylation. Conversely, overexpressing Arg2 reverses sorafenib-induced ferroptosis, which is inhibited by Akt inhibitor. Thus, inhibition of Arg2 can impair the anticancer efficiency of sorafenib in MM cells. These results suggests that Arg2 functions as a suppressor of ferroptosis through activating the Akt/GPX4 signaling pathway in melanoma cells (Yu et al., 2022).

### 4.6 The role of microRNAs in regulating ferroptosis in MM

MicroRNAs (miRNA) have been reported to regulate ferroptosis in MM (Luo et al., 2018). miR-137 inhibits ferroptosis by directly targeting SLC1A5, a glutamine transporter, in MM cells. Overexpression of miR-137 suppresses SLC1A5, resulting in decreased uptake of glutamine and accumulation of MDA (Luo et al., 2018). Silencing miR-137 increases the sensitivity of MM cells to RSL3-and erastin-induced ferroptosis, while knockdown of miR-137 boosts the antitumor efficiency of erastin by promoting ferroptosis (Luo et al., 2018). These results indicate that miR-137 negatively regulates ferroptosis through inhibition of glutaminolysis, highlighting a potential therapeutic target for MM (Luo et al., 2018). miR-9 has been shown to function as an inhibitor of ferroptosis through directly binding glutamicoxaloacetic transaminase 1 (GOT1), which catalyzes the conversion of glutamate to α-ketoglutarate. Overexpressed miR-9 directly binds to and suppresses GOT1, leading to inhibition of erastin- and RSL3-induced ferroptosis (Zhang et al., 2018). Silencing miR-9 promotes iron accumulation and LPO and increases the sensitivity of MM cells to erastin and RSL3, while inhibition of glutaminolysis abrogates anti-miR-9 mediated ferroptosis (Zhang et al., 2018). These findings reveal the important role of miRNA in regulating ferroptosis in MM. Increased expression of the lncRNA AGAP2-AS1 promotes tumorigenesis by promoting ferroptosis evasion through increased SLC7A11 mRNA stability by the m^6^A modification in an IGF2BP2-dependent manner in MM (An et al., 2022). AGAP2-AS1 functions as an oncogene in MM, and its increased expression is known to be significantly associated with a poor prognosis (An et al., 2022). Silencing AGAP2-AS1 inhibits melanocytes growth through increasing erastin-mediated ferroptosis, which was reversed by the ferroptosis inhibitor Ferrostatin-1 (An et al., 2022).

## 5 Induction of ferroptosis as a novel approach to treat melanoma

Therapy-resistant cancer cells exhibit metabolic states, including but not limited to increased PL-PUFAs associated with EMT, that influence the vulnerability of cancer cells to the induction of ferroptosis. Melanoma cells exhibit a vulnerability to ferroptosis, which may possibly result from increased accumulation of PUFAs and low levels of GSH (Tsoi et al., 2018). Intriguingly, drug-tolerant persister cancer cells or mesenchymal cancer cells resistance to therapyare vulnerable to ferroptosis, which is likely to be associated with their dependence on GPX4 function (Hangauer et al., 2017; Viswanathan et al., 2017). BRAFi treatment promotes dedifferentiation of MM cells, leading to increased susceptibility of these MM cells to ferroptosis (Tsoi et al., 2018). In this part, we emphasize the emerging types of small molecules that are capable of inducing ferroptosis pathways by boosting the antitumor activity of BRAFi, immunotherapy, and ferroptosis inducers and delineating their beneficial effects in treating MM. Finally, we summarize the application of nanosensitizer-mediated unique dynamic therapeutic strategies and ferroptosis-based nanodrug targeting strategies as therapeutic options for MM.

### 5.1 Small molecules targeting ferroptosis pathways

#### 5.1.1 Boosting the antitumor activity of BRAFi

Targeted agents for BRAF-mutant MM have significantly improved the overall survival of patients with MM (Proietti et al., 2020; Poulikakos et al., 2022; Zhong et al., 2022). BRAFi as well as five combinations of BRAFi plus an additional agent(s) have been used to manage cancers such as melanoma (Poulikakos et al., 2022). However, since acquired resistance to targeted therapy is common and most patients show resistance to BRAF inhibitors (BRAFi), overcoming resistance to BRAFi is an unmet need and strategies to manage drug resistance are urgently required. Ferroptosis-inducing regimens have been shown to be one approach to overcome resistance to targeted therapy (Zhang et al., 2022). The AXL inhibitor (AXLi) BGB324 increases the sensitivity of A375 cells to the BRAF inhibitor (BRAFi) vemurafenib by stimulating ferroptosis and inhibiting autophagy, suggesting that a combination of AXLi with standard therapy is a promising approach to boost therapeutic outcomes in metastatic MM (Nyakas et al., 2022). Lipid metabolic reprogramming, one of the hallmarks of MM cells, has been shown to contribute to tumor resistance to targeted therapy (Vergani et al., 2022). MM cells show enhanced levels of stearoyl-CoA desaturase 1 (SCD1) and acetyl-CoA acetyltransferase 2 (ACAT2), resulting in resistance to BRAFi (Vergani et al., 2022). Thus, silencing ACAT2 can impair resistance to PLX4032. The acyl-CoA cholesterol acyl transferase (ACAT/SOAT) inhibitor avasimibe shows antiproliferative effects by boosting the antitumor activity of PLX4032/vemurafenib through induction of ferroptosis in MM (Vergani et al., 2022). High expression of AXL promotes the switch from an epithelial to a more aggressive mesenchymal phenotype in melanoma, limiting treatment efficacy. In this regard, the AXL inhibitor (AXLi) BGB324 can increase the sensitivity of A375 cells to the BRAF inhibitor (BRAFi) vemurafenib by inducing ferroptosis and apoptosis and inhibiting autophagy (Nyakas et al., 2022). *C. zeylanicum* essential oil (CINN-EO) has been shown to induce inhibition of cell growth by inducing ferroptosis. CINN-EO promotes the anti-melanoma effect of the mitochondria-targeting antineoplastic drugs tamoxifen and the BRAFi dabrafenib (Cappelli et al., 2023). Recent researches have revealed that sorafenib, the first multi-tyrosine kinase inhibitor to treat differentiated thyroid carcinoma, unresectable HCC, and advanced-stage renal cell carcinoma, induces ferroptosis (Hadian and Stockwell, 2020; Chen et al., 2021). Sorafenib also increases the sensitivity of MM cells to vemurafenib through inducing ferroptosis (Tang et al., 2020).

#### 5.1.2 Boosting cancer immunotherapy

Over the last decade, anti-programmed cell death 1 (PD1) antibodies (Abs) and ICIs, in combination with or without another drugs such as anti-CTLA-4 Abs, have been widely used for the treatment of advanced and metastatic melanoma (Larkin et al., 2015; Larkin et al., 2019). Since anti-PD1 Abs can be used for treating advanced MM even without BRAF mutations, anti-PD1 Ab-based regimens to treat advanced MM have recently been developed (Fujimura et al., 2020). Through abrogation of CTLA-4 and PD-1, ICIs are currently the standard reference therapy in patients with advanced MM. Recent studies have shown that targeting Wnt/β-catenin signaling can boost the efficacy of anti-PD-1 immunotherapy in MM by exacerbating ferroptosis via regulation of MITF (Wang Y. et al., 2022). Induction of ferroptosis significantly inhibits Wnt/β-catenin signaling in MM, and the activation of Wnt/β-catenin signaling enhances the transcription of MITF, resulting in upregulation of the downstream peroxisome proliferator-activated receptor gamma coactivator 1-alpha (PGC1α) and stearoyl-CoA desaturase (SCD1), which suppresses LPO to inhibit ferroptosis (Wang H. et al., 2022). Pharmacological inhibition of β-catenin by ICG001 promotes MM cell ferroptosis by increasing LPO both *in vitro* and *in vivo*. Pharmacologically inhibiting of β-catenin or MITF boosts antitumor activity of anti-PD-1 immunotherapy by promoting ferroptosis in a preclinical xenograft tumor model (Wang M. et al., 2022). These results suggest that targeting the Wnt/β-catenin-MITF pathway is a promising strategy to boost the efficacy of anti-PD-1 immunotherapy through potentiation of ferroptosis in MM.

#### 5.1.3 Boosting the antitumor activity of ferroptosis inducers

Nrf2 is efficiently activated in resistant MM cells, leading to upregulation of the early ferroptosis marker ChaC glutathione-specific gamma-glutamylcyclotransferase 1 (CHAC1) and the aldo-keto reductase AKR1C1 ÷ 3 that degrades 12/15-LOX-mediated production of lipid peroxides to induce ferroptosis resistance. However, medroxyprogesterone (MPA), a pan-inhibitor of AKR1C1 ÷ 3, inhibits AKR activity/expression to completely enhance the susceptibility of resistant melanoma cells to ferroptosis induction (Gagliardi et al., 2019). These results indicate that the use of ferroptosis inducers coupled to AKR inhibitors can serve as a new regimen to efficiently kill MM cells.

#### 5.1.4 Small molecules inducing ferroptosis

DET and DETD-35 trigger ferroptosis in PLX4032-sensitive (A375) and PLX4032-resistant (A375-R) BRAFV600E melanoma cells through inhibiting GPX4 via non-covalent binding (Chang et al., 2022). While lnc NEAT1 is known to be upregulated in melanoma, downregulation of lnc NEAT1 induces ferroptosis through weakening of the direct binding to SLC7A11, indirectly resulting in inhibiting GPX4 and inducing ferroptosis. Gambogenic acid induces ferroptosis through downregulation of lnc NEAT1 (Wang S. et al., 2022). Gambogenic acid significantly inhibits migration, invasion, and EMT in MM cells through inducing ferroptosis via the p53/SLC7A11/GPX4 signaling pathway (Wang et al., 2020). Hyperforin inhibits cell growth by inducing apoptosis, autophagy, and ferroptosis through a reduction in GPX4 expression (Cardile et al., 2023). RSL3-induced cell death is fully reversed by ferrostatin-1 in A375 melanoma cells, indicating their high susceptibility to ferroptosis (Tyurina et al., 2023). RSL3 induces ferroptosis through inhibition of Wnt/β-catenin signaling (Wang Y. et al., 2022). Gallic acid increases the sensitivity of A375 cells to low-level laser through induction of apoptosis and ferroptosis (Khorsandi et al., 2020). Nobiletin exerts antitumor activity through inducing ferroptosis via downregulating GSK3β-mediated Keap1/Nrf2/HO-1 signaling pathway in human MM cells (Feng et al., 2022). Aridanin exhibits antitumor activity by inducing ferroptosis (Mbaveng et al., 2020). TX1-85-1, a small-molecule inhibitor targeting the ErbB3 signaling pathways, sensitizes melanomas to ferroptosis activators (Leu et al., 2022). Mycalols trigger ferroptosis through downregulation of GPX4 and upregulation of NCOA4 (Riccio et al., 2021). BAY 87-2243 stimulates autophagosome formation, mitophagy, and ROS generation, leading to the combined activation of necroptosis and ferroptosis (Basit et al., 2017).

### 5.2 Nano-based medicines targeting the ferroptosis pathway

#### 5.2.1 Nanomaterial-based dynamic therapy to induce ferroptosis for melanoma treatment

Unique dynamic therapies mediated by nanosensitizers represent an effective tactic for the treatment of deep solid tumors (Sun et al., 2023). Multiple innovative dynamic-therapy strategies using nanosensitizers with unique physicochemical properties that respond to highly penetrating excitations to kill various deep-seated malignant tumors have been recently developed, including sonodynamic therapy (SDT) (Guo J. et al., 2022; Nowak et al., 2022; Yang et al., 2023), X-ray-induced photodynamic therapy (PDT) (Wang et al., 2016; Jiang et al., 2023; Kolarikova et al., 2023), and chemodynamic therapy (CDT) (Yu et al., 2021; Cao et al., 2022). EDN3-CPNPs carrying iron (EDN3-CPNPs) can boost the cancer cell-killing efficacy of ferroptosis-assisted CDT to over 80% at higher doses (Jasim and Gesquiere, 2019). Ir-PBT-BPA, which is formed by light irradiation of cyclometalated Ir(III) complexes, can produce superoxide anion radicals and singlet oxygen, which induce cell death by a combination of ferroptosis and ICD. Ir-PBT-BPA induces the depletion of regulatory T cells and immune response of CD_8_^+^ T cells while increasing the number of effector memory T cells, thereby achieving long-term antitumor immunity (Wang L. et al., 2023). PPIX-PSilQ NPs induce cell death through the induction of ferroptosis, as evidenced by increased production of ROS and LPO (Vadarevu et al., 2021). Mild photothermal therapy (mPTT, 42°C–45°C) has shown promising potential in tumor therapy with better biological effects and less side effects (He et al., 2023). Fe@OVA-IR820 induces Fe^3+^-dependent ferroptosis-triggered ICD, which releases endogenous neoantigens and DAMPs that work synergistically with the exogenous antigen ovalbumin (OVA) to provoke an immune response. The photothermal effect of near-infrared irradiation further amplifies these immune responses (Ma G. et al., 2023). The increased recruitment and infiltration of T cells suppresses the primary tumor. The combination of Fe@OVA-IR820 nanovaccine with CTLA-4 checkpoint blockade significantly boosts anticancer immunity and halts the growth of distal simulated metastases (Ma S. et al., 2023). PDT promotes the sensitivity of MM cells to PTT by hampering the tumor microenvironment, whereas PTT-induced heat increases blood flow, improves the supply of oxygen, and boosts the therapeutic effects of PDT (Kong and Chen, 2022). The combination of PDT with PTT has been selected as a tumor-ablation regimen in various cancer indications. Au NRs/Cur/UCNPs@PBE activate both ferroptosis and apoptosis to achieve synergistic PDT/PTT. Au NR/Cur/UCNP@PBE-mediated combined PTT with PDT shows greater antitumor efficacy than other single treatments *in vivo* (Zhong et al., 2021).

#### 5.2.2 Nanomaterials that induce ferroptosis for melanoma treatment

Nanotechnology provides new opportunities for tumor therapy through the induction of ferroptosis (Fei et al., 2020; Sepand et al., 2020; Wang G. et al., 2021; Li et al., 2021; Luo et al., 2021; Wu and Zhang, 2021; Zheng et al., 2021; Fernández-Acosta et al., 2022; Shi et al., 2022; Zeng et al., 2022; Liu K. et al., 2023; Liu Q. et al., 2023; Ma S. et al., 2023). This part presents approaches for harnessing nanomaterials to induce ferroptosis and kill cancer cells. MM.GW486, an exosome inhibitor with a ferroptosis inducer (iron ion), has been used in a hyaluronic acid (HA)-based nanoplatform (HGF-NPs). HGF-NPs inhibit exosomal PD-L1 and immunostimulation. HGF-enhanced tumor cellular ferroptosis. The combination of HGF with anti-PD-L1 immunotherapy has been shown to effectively inhibit MM metastasis (Wang Y. et al., 2021). The alpha melanocyte-stimulating hormone (αMSH) targets melanocortin-1 receptor (MC1-R), a surface receptor that is expressed on malignant MM cells (Miao et al., 2007). Kim et al., (2016) developed silica-based ultrasmall αMSH-PEG-C′ dots with a 6-nm diameter, in which silica-based particles with a Cy5-encapsulated fluorescent core and polyethylene glycol (PEG) coating and αMSH-modified exterior. These αMSH-PEG-C′ dots inhibited tumor growth by inducing ferroptosis. The inhibitor of ferroptosis, liproxstatin-1reverse αMSH-PEG-C′-mediated tumor growth inhibition, in tumor xenografts in mice (Kim et al., 2016). Jiang and others constructed a photosynthetic microcapsules (PMCs), which encapsulate cyanobacteria and upconversion nanoparticles in alginate microcapsules and are driven by external near-infrared photons. The combination of PMCs with X-rays induced ferroptosis in MM cells and xenografts, providing evidence for the development of lipid peroxidation, GPX4 suppression, Fe^2+^ release, and GSH reduction (Jiang et al., 2022). Consequently, the combined treatment overcame the intrinsic and acquired resistance to MM, thereby inhibiting metastases and improving the survival rate of melanoma-bearing mice (Jiang et al., 2022). Li et al., (2023) constructed a nanoscale metal-organic framework (MOF) Cu-BTC as a carrier and loaded diethyldithiocarbamate (DDTC) through coordination interactions, i.e., Cu-BTC @DDTC. Cu-BTC@DDTC shows anticancer potential by inducing ferroptosis, especially in combination with low-dose cisplatin (Li et al., 2023). Upregulated miR-21-3p promotes IFN-γ-mediated ferroptosis by potentiating LPO in MM. miR-21-3p increases the sensitivity to ferroptosis by directly targeting thioredoxin reductase 1 (TXNRD1) to increase lipid ROS generation. Overexpressed miR-21-3p acts synergistically with anti-PD-1 antibody to promote ferroptosis in MM. miR-21-3p-loaded gold nanoparticles have been shown to boost the efficacy of anti-PD-1 antibodies without causing prominent side effects in a mouse model (Guo W. et al., 2022).

## 6 Conclusion and perspectives

This review presents the recent progress in our understanding of the role of ferroptosis in melanoma. Metabolic switch, GPX4-dependent persister state, cellular dedifferentiation status, and ferroptosis evasion dictate the sensitivity or resistance of melanoma to ferroptosis. Emerging evidence has confirmed the role of microRNAs in the regulation of ferroptosis in MM. Due to accumulation of PUFAs and low levels of GSH, MM cells show vulnerability to ferroptosis. Induction of ferroptosis is generally considered to be an effective approach to induce cell death in therapy-resistant MM cells. Small molecules targeting ferroptosis pathways can boost the antitumor activity of BRAFi and cancer immunotherapy. Meanwhile, nano-based medicines can induce the ferroptosis pathway in MM through dynamic therapy and nanomaterials that induce ferroptosis. However, these findings also highlight the need to understand the factors dictating the sensitivity of MM cells to ferroptosis, considering the substantial patient-to-patient variability in drug resistance mechanisms. In this scenario, identification of new molecular markers that correlate the phenotype of MM cells with vulnerability to induction of ferroptosis and identifying other regulators that control the acquisition of this phenotype should be the research directions in future studies. Relatively little is known about the process through which ferroptosis orchestrates diverse cellular events, and future research in MM should undoubtedly focus more on delineating the roles of additional potential regulators of ferroptosis, including hippo signaling, transsulfuration, mevalonate synthesis pathways, and iron metabolism. How current findings replicate across multiple MM models. Although some ferroptosis inducers have shown a safe profile in mice, a critical requirement is to translate these findings to human patients with MM to understand whether the same conclusions can be drawn for human patients as well. In addition, several small molecules have been identified as ferroptosis inducers in other cancers. Thus, these drugs may be repurposed for the treatment of MM. Therefore, continued exploration of the roles of ferroptosis in MM, and the relationship between ferroptosis and MM will facilitate the discovery of novel therapeutic strategies for MM.
